# Reviews on Mechanisms of *In Vitro* Antioxidant Activity of Polysaccharides

**DOI:** 10.1155/2016/5692852

**Published:** 2015-11-22

**Authors:** Junqiao Wang, Shuzhen Hu, Shaoping Nie, Qiang Yu, Mingyong Xie

**Affiliations:** State Key Laboratory of Food Science and Technology, Nanchang University, 235 Nanjing East Road, Nanchang, Jiangxi 330047, China

## Abstract

It is widely acknowledged that the excessive reactive oxygen species (ROS) or reactive nitrogen species (RNS) induced oxidative stress will cause significant damage to cell structure and biomolecular function, directly or indirectly leading to a number of diseases. The overproduction of ROS/RNS will be balanced by nonenzymatic antioxidants and antioxidant enzymes. Polysaccharide or glycoconjugates derived from natural products are of considerable interest from the viewpoint of potent *in vivo* and *in vitro* antioxidant activities recently. Particularly, with regard to the *in vitro* antioxidant systems, polysaccharides are considered as effective free radical scavenger, reducing agent, and ferrous chelator in most of the reports. However, the underlying mechanisms of these antioxidant actions have not been illustrated systematically and sometimes controversial results appeared among various literatures. To address this issue, we summarized the latest discoveries and advancements in the study of antioxidative polysaccharides and gave a detailed description of the possible mechanisms.

## 1. Introduction

Polysaccharide is a high molecular weight polymer, consisting of at least ten monosaccharides mutually joined by glycosidic linkages. The glycosyl moiety of hemiacetal or hemiketal, together with the hydroxyl group of another sugar unit, formed the glycosidic linkages [1]. Unlike protein and nucleic acid, the structure of polysaccharide is far more complicated based on the differences in composition of monosaccharide residues, glycosidic linkages, sequence of sugar units, degrees of polymerization, and branching point. Apart from those, other factors, such as differences of cultivars, origins, and batches, or even extraction methods and fraction procedures are evidenced to have significant influence on the physicochemical and structural properties of polysaccharides. Owing to the rapid development of modern analytical techniques, the identification of polysaccharide structures is becoming more and more feasible and convenient.

In recent years, researches have confirmed that polysaccharides from natural products possess wide-ranging beneficial therapeutic effects and health-promoting properties. Specifically, seaweed-derived polysaccharides, such as alginate, fucoidan, carrageenan, laminaran, and agar [2], are widely distributed in biomedical and biological applications [3–8], for example, tissue engineering, drug delivery, wound healing, and biosensor due to their biocompatibility and availability. Fungal polysaccharides, derived from* G. frondosa*,* L. edodes*, oyster mushroom, as well as* Ganoderma*,* Flammulina*,* Cordyceps*,* Coriolus*, and* Pleurotus*, and so forth, are demonstrated to have multiple bioactivities [9–15], including immunomodulating, anticancer, antimicrobial, hypocholesterolemic, and hypoglycemic effects. Bacterial polysaccharides, including extracellular polysaccharides, loosely associated with bacterium, capsular polysaccharides, tightly bound to bacteria surface, and lipopolysaccharides, always anchored to cell surface by lipid, are nontoxic natural biopolymers and provide extensive applications in areas such as pharmacology, nutraceutical, functional food, cosmeceutical, herbicides, and insecticides [16–18]. Consequently, there is growing interest in further pursuing the potential bioactivities of diverse polysaccharides. In particular, most of these polysaccharides emerged as an important agent of antioxidants, both* in vitro* and* in vivo*. Recently, the polysaccharide is reported as a kind of effective free radical scavenger and antioxidants, playing a critical role in protecting against oxidation damage in living organisms. On the other hand, many diseases, such as asthma, chronic obstructive pulmonary disease, inflammation, diabetes, myocardial infarction, and cardiovascular diseases, are reported to associate with oxidative stress [19–23]. This paper aims to review the recent advancements in analyzing antioxidative polysaccharides and summarize the possible mechanisms so as to better utilize the biopolymer.

## 2. Types of* In Vitro* Antioxidant Assays

Many different* in vitro* models have been introduced to evaluate the antioxidant activities so as to assess an antioxidant that would be useful for food and biological system [24, 25]. Generally, methods for determining antioxidant activities could be classified into two major groups: hydrogen atom transfer (HAT) based methods and single electron transfer (SET) based methods according to their reaction mechanisms [26, 27]. The HAT based methods usually measure the ability of quench free radical by hydrogen donation, that is, oxygen radical absorbance capacity (ORAC), total radical-trapping antioxidant parameter (TRAP), inhibition of induced low-density lipoprotein (LDL) oxidation, total oxyradical scavenging capacity assay, and so forth. On the other hand, SET based methods detect the ability of transferring one electron to reduce any compound, including metals, carbonyls, and radicals, and result in a change in color when this compound is reduced, such as Trolox equivalence antioxidant capacity (TEAC) assay, ferric ion reducing antioxidant power (FRAP) assay, and 2,2-diphenyl-1-picrylhydrazyl radical (DPPH) scavenging. Other assays, for example, superoxide radical scavenging, hydrogen peroxide scavenging, and singlet oxygen quenching, evaluate the scavenging ability for oxidants.

## 3. Factors That Influence the Antioxidant Activity of Polysaccharides

Recently, natural materials are proved to be a highly promising source of antioxidants, since a wide range of bioactive constituents derived from them, such as flavonoids, polyphenols, sterols, peptides [28], polysaccharides, and others [29–33], have been reported to possess strong antioxidant abilities. Screening bioactive compounds from natural materials based on antioxidant potentials is widely adopted at present. Ray et al. [34] employed DPPH-scavenging-guided fractionation with silica gel column chromatography to separate potent fractions from methanolic extract of* Aloe vera* L. gel. Hossain et al. [35] obtained three fractions with high activities from the marjoram based on the results of DPPH and ferric ion reducing antioxidant power assays.

Previously, polysaccharides and polysaccharide-complex extracted from many natural sources, including higher plants, fungi, marine flora, and fauna, are of considerable interest from the viewpoint of multipharmacological activities and potential advancement towards food, nutraceuticals, and pharmaceutical industry [9, 36–39]. However, despite the great antioxidant potentials of polysaccharides exerted, their underlying mechanism is not systematically elucidated. As a result, the following sections summarize the current understanding of possible antioxidant mechanisms of polysaccharides.

### 3.1. Polysaccharide Conjugates

Natural polysaccharides do not always exist singly but conjugate with other components, such as amino acid, protein, lipids, and nucleic acids residues, and sometimes the polysaccharide conjugates act as a whole in isolation [40]. For example, cereal polysaccharides were reported to be associated with a certain amount of phenolic compounds [41] and tea polysaccharides were mostly glycoconjugates in which a protein carries carbohydrate chain covalently linked with a polypeptide backbone [42]. The formation of polysaccharide-polyphenol conjugates would be mediated by either H-bonding or hydrophobic interactions, and for polysaccharide-protein conjugates it may be by the existence of hydrophobic cavities and crevasses [43].

Several studies have postulated that the protein or peptide moiety in polysaccharide is responsible for part of radical scavenging effect. As mentioned in a report by Liu et al. [44], the content of protein in polysaccharide extracts appeared to contribute a direct scavenging effect on superoxide and hydroxyl radicals. Lentinan and* Schizophyllum* with only trace amount of protein exhibited negligible scavenging effect towards superoxide radicals, whereas polysaccharide-protein complexes extracted from mushrooms such as* Ganoderma* and* Grifola*, with lower polysaccharide/protein ratios, were more favorable for the scavenging function. Similarly, Huang et al. [45] demonstrated that the protein-free fractions (P_1/5_ and P_2/5_) separated from fermentation medium of* Cordyceps sinensis* did not show any antioxidant properties, while the fraction (P_5_) with high amount of protein exhibited remarkable activity. In their previous study, TEAC value was discovered to correlate with the protein content of exopolysaccharide fractions as well [46]. Liu et al. [47] also supposed that the superoxide radical scavenging effect of crude polysaccharide from* Athyrium multidentatum* (Doll.) Ching (CPA) depended on the amount of peptides presented as a form of polysaccharide-peptide complex in CPA. Moreover, the antioxidant activity of polysaccharide-protein complexes from three mushrooms,* G. frondosa*,* Coriolus versicolor*, and* L. edodes*, attained by ultrasound-assisted extraction was generally higher than conventional hot-water method, which probably attributed to the fact that ultrasound treatment resulted in an increase of protein content in polysaccharides [48]. Zhang et al. [49] isolated three extracts (EXT-A, EXT-B, and EXT-C) from paddlefish cartilage by single alkali method, microwave-assisted alkali without or with deproteinization, respectively. Result showed that EXT-B, containing predominantly protein (87.9%), exhibited noticeable antioxidant potentials with TEAC value of 118.5 *μ*mol Trolox/g sample and FRAP value of 107.7 *μ*mol Fe^2+^/g sample, while EXT-C with total sugar content of 99.0% showed little activity, indicating that the protein constituents in the extracts play a dominant role. The further amino acid composition analysis showed that EXB-1 was abundant in tyrosine, glycine, and glutamic acid, and this investigation was performed to clarify the speculation, since the antioxidant action of protein or peptide molecules has been proved to be related to their amino acids, such as tyrosine, methionine, histidine, lysine, and tryptophan, which were capable of donating protons to electron-deficient radicals [50–52].

On the contrary, in some cases, lack of correlation between the protein content and the FRAP value was also noticed, possibly attributing to the functional groups of protein (such as -SH) which are less sensitive to FRAP assay [46].

Phenolic compounds, especially phenolic acids, play an important role in the overall radical scavenging ability of xylans and xylooligosaccharides from the wheat bran [53, 54]. Hromádková et al. [55] pointed out that both protein and phenolic compounds contributed to the radical scavenging effects of xylans, and the protein-free fraction displayed the highest hydroxyl radical scavenging ability indicating the distinct role of phenolic acids. A study [56] revealed that the antioxidant activities of all polysaccharide fractions from three mushrooms (*L. edodes, G. frondosa*, and* T. versicolor*) were significantly correlated with the total phenolic and protein content according to three* in vitro* assessments, including TEAC, FRAP, and ferrous ion chelating activity assay. However, no significant correlation was observed between the total sugar content and any of tested antioxidant assays. The results were similar to a study carried out by Wang et al. [57] that the neutral content was not apparently correlated with DPPH and FRAP antioxidant actions of polysaccharides from oolong tea. Furthermore, purified polysaccharide fractions, free of phenolics and proteins, hardly showed significant antioxidant activities. Indeed, polysaccharide-polyphenol residues have been demonstrated to have noticeable antioxidant functions in many reports. Li et al. [58] found no statistical difference in scavenging linoleic acid radicals between the polysaccharides from* Lycium barbarum* fruits and the positive control (BHT). The coupled oxidation of *β*-carotene and linoleic acid developed free radicals, which oxidize unsaturated *β*-carotene molecules, leading to the discoloration of the system. In this model, the proposed mechanism in hindering *β*-carotene oxidation could be attributed to the polyphenolic-associated polysaccharide neutralizing the free radicals. In DPPH radical scavenging assay, the polysaccharide showed pronounced antioxidant ability as well, possibly attributing to polyphenolic-associated polysaccharide fraction formed between high molecular weight phenolics and polysaccharides.

However, not all the conjugated moiety of polysaccharides was responsible for antioxidant power. After removing polyphenols, the tea polysaccharide conjugate from low grade green tea was found to possess strong antioxidant properties based on the results of free radical scavenging and lipid peroxidation inhibitory effect [59]. Likewise, Wang et al. [60] evidenced that the DPPH radical scavenging effect of another tea polysaccharide fraction (TPS1) was beyond 90%, close to that of ascorbic acid, although both of the protein and polyphenol content were relatively low in TPS1, suggesting other factors such as carboxyl group other than polyphenol compounds that are of concern. In order to determine the molecular interactions between tea polyphenols and oat *β*-glucan, Wu et al. [61] prepared complex and physical mixture of oat *β*-glucan and tea polyphenols, further using four* in vitro* antioxidant evaluations (DPPH radical, hydroxyl radical, superoxide radical, and reducing power) to compare the activity among tea polyphenols, *β*-glucan, their complex, and physical mixture. Results showed that the complex had the strongest effect against superoxide radical, whereas the mixture had the strongest hydroxyl radical scavenging effect in the concentration of 0.5–2.5 mg/mL. With regard to reducing power assay, no synergistic effect was found between tea polyphenols and *β*-glucan, but it was observed in DPPH scavenging assay when *β*-glucan was combined with tea polyphenols at low concentration (<0.05 mg/mL). However, when tea polyphenol was used at a high concentration (0.09 mg/mL), it was changed to antagonistic effect in scavenging DPPH radical. The inconsistent antioxidant outcomes of tea polyphenols and oat *β*-glucan complex might be dependent on its structure and provided dose, as well as the strong hydrogen bonds between them.

Ferulic acid, a kind of phenolic acid and a strong antioxidant, was shuttled to wall matrices via attachment to structural polysaccharides. Feruloylation, in certain cases, occurs on the arabinose or galactose side chains of pectin polysaccharides and influences their chemical properties. The attachment of ferulic acid is covalently via an ester linkage formed between carboxylic acid group and the primary hydroxyl at carbon-5 position of *α*-L-arabinofuranosyl residues [62, 63]. Some researchers [64, 65] obtained feruloyl oligosaccharide (FH) released from wheat bran insoluble dietary fiber by xylanases and found that FH could inhibit 91.7% of erythrocyte hemolysis induced by peroxyl radicals and retard the hemolytic initiation for more than 120 min under* in vitro* condition at a concentration of 4 mg/mL.

Therefore, the content of total phenolic or protein compounds conjugated in the polysaccharide extracts might explain their high antioxidant potentials.

### 3.2. Polysaccharide Mixture

In many reports, crude polysaccharide extracts exhibited notable antioxidant activity, but after further fractionation, the final purified polysaccharide showed moderate or low activity. It seemed that other antioxidant substances contained in the crude polysaccharide extract, such as pigments, flavones, peptide, protein, and polyphenol, might contribute to the antioxidant activity [13, 66]. Wang et al. [67] investigated the role of tea polyphenol (EGCG) in crude polysaccharide extracts from tea leaves (TPS) in the view of antioxidant ability. Results showed that the crude TPS exhibited strong antioxidant functions, whereas the further purified TPS fractions were hardly effective. But in the presence of EGCG, the reducing power and DPPH radical scavenging ability of TPS fractions were obviously enhanced. Meanwhile, the same results were also observed in dextran-EGCG system, indicating EGCG caused a synergistic increase in the antioxidant activity and tea polyphenol was the major antioxidant in the crude TPS. Mu et al. [68] illustrated the existence of protein and pigment would influence the scavenging effect of both water-soluble and alkali-soluble crude polysaccharides from* Inonotus obliquus*. Wei et al. [69] purified an acidic polysaccharide from* Prunella vulgaris* Linn., of which scavenging abilities against DPPH and hydroxyl radical were significantly lower than crude polysaccharide, possibly ascribed to other antioxidants, such as flavones and pigments contained in the crude polysaccharide extracts. Lin et al. [70] compared the antioxidant properties of different polysaccharide fractions isolated from* Lycium barbarum* Linnaeus, including crude polysaccharide (CP), crude extract of polysaccharide (CE), deproteinated polysaccharide (DP), and deproteinated and dialyzed polysaccharide (DDP), as well as four purified fractions (one neutral and three acidic polysaccharides, named as LBPN, LBPa1, LBPa2, and LBPa3, resp.). In their study, it was suggested that the inhibition effect of superoxide and hydroxyl radical by hydroxyl groups in polysaccharides was minor due to lacking phenolic-type structure which was essential for scavenging free radicals. Many other factors, such as molecular weight, galacturonic acid, and other chemical components in polysaccharide fractions, were also supposed to play a role in their antioxidant activities. Crude and purified polysaccharide, obtained from* G. atrum*, was compared in terms of DPPH scavenging ability and self-oxidation of 1,2,3-phentriol. Although the high concentration of purified polysaccharide, PSG-1, showed noticeable antioxidant ability, it was much lower than the crude polysaccharide, probably attributing to other constituents contained in crude polysaccharides extracts, such as proteins, amino acids, peptides, cellulose, phytosterol, ascorbic acid, thiamine, nucleotide, nicotinic acid, organic acids, and microelements [71].

### 3.3. Polysaccharide Chelating Metal

It is worth noting that one mechanism of antioxidant activity is to inhibit the generation of free radicals by chelating ions such as ferrous and copper instead of directly scavenging them. Transition metal ions could catalyze the generation of extremely reactive hydroxyl radicals from superoxide and hydrogen peroxide, known as Fenton reaction (Fe^2+^ + H_2_O_2_ → Fe^3+^OH^−^ + OH^·^), especially ferrous ion, which is the most effective prooxidant in the food system [72]. Two polysaccharide fractions (GAPS-1 and SAPS-1) from* A. barbadensis *Miller were isolated and purified. The hydroxyl radical scavenging activity of GAPS-1 was significantly higher than SAPS-1. Meanwhile, GAPS-1 had a higher chelating ability against ferrous ion, indicating that the chelating effect might impart polysaccharides capable of antioxidant potentials [73]. A similar correlation was also revealed by Li et al. [74] investigating an extracellular polysaccharide from* N. commune*.

Generally, the structure of compounds containing more than one of the following functional groups, that is, -OH, -SH, -COOH, -PO_3_H_2_, -C=O, -NR_2_, -S-, and -O-, is in favor of chelating ability [75]. Therefore, presence of uronic acid and sulfate groups appeared to be essential in demonstrating the chelating ability of polysaccharides. Chang et al. [76] illustrated that the larger the content of galacturonic acid in polysaccharide, the higher the ability of chelating ferrous ion. Fan et al. [66] fractionated four polysaccharides from the leaves of* Ilex latifolia *Thunb. by DEAE cellulose-52 chromatography (ILPS1, ILPS2, ILPS3, and ILPS4) with ILPS4 having the highest contents of sulfuric radical (3.7%) and uronic acid (23.2%). Results showed that IC_50_ of ferrous chelating activity for ILPS4 was 1965 ± 8.1 *μ*g/mL, while for other fractions the abilities were 4.7%, 11.3%, and 46.7%, respectively. This observation confirmed that the chelating effect might partly be due to the presence of functional groups such as carboxyl group and sulfuric radical in the polysaccharide structure. However, the ferrous ion chelating effect of a carboxymethylated polysaccharide (C-GLP) from* G. lucidum *was weak as compared to EDTA [77]. The reason was probably attributed to its structural features unsuitability for chelating metal ion, as the chelating ability of ferrous was dependent upon hydroxyl numbers and the hydroxyl substitution in the ortho position [78].

### 3.4. Metal Ions-Enriched Polysaccharide

Selenium (Se) is an essential trace element for nutrition of a capital importance in the human biology. Se does not directly act as a ROS/RNS scavenger but is a cofactor of selenoprotein, for example, glutathione peroxidase, which exerts various antioxidant activities* in vivo*. Confirmed by FT-IR and NMR spectra, the selenylation modification by H_2_SeO_3_/HNO_3_ method predominantly happened at the C-6 position of polysaccharides and a distinct decrease of molecular weight was also induced due to the acid environment of selenized reaction. Additionally, it was proposed that the combination of Se in polysaccharides was possibly in the form of selenyl group (-SeH) or selenoacid ester [79]. Wei et al. [80] synthesized a series of selenylated polysaccharide from* Radix hedysari* (Se-RHP), the content of Se ranging from 1.04 to 3.29 mg/g and the molecular weight decreasing from 62.7 kDa to 27.7 kDa, which showed better scavenging activity and reducing power in contrast to the native RHP.

Likewise, Se-containing derivatives from* Artemisia sphaerocephala* [81] and* Potentilla anserina *L. [82] have been acknowledged to improve the antioxidant activity compared to the native polysaccharides. The proposed mechanism might be involved in changes of conformation structure of polysaccharides and emerged the increasing amount of hydroxyl group, resulting in an influence on the antioxidant activity.

Further analysis on polysaccharide obtained from Se-enriched materials confirmed the important role of Se in enhancing the antioxidant potentials of polysaccharides. As evidenced by the results of Yu et al. [83], polysaccharide from Se-enriched green tea presented significant higher antioxidant capacity than that from regular green tea. In addition, all polysaccharides isolated from Se-enriched* G. lucidum* were more effective on attenuating the production of superoxide radicals [84]. Mao et al. [85] revealed that although there was no significant difference of polysaccharide content and molecular weight of each Se-enriched* G. frondosa* polysaccharide (Se-GP) fraction and the corresponding GP, except for the Se content, Se-GPs were a more effective scavenger (against DPPH, ABTS, and hydroxyl radicals), especially for hydroxyl radical, reaching 71.32% at a concentration of 2 mg/mL. On the other hand, selenium-polysaccharide synthesized by adding selenium chloride oxide (SeCl_2_O) also exhibited a higher total antioxidant capacity, superoxide radical, and hydroxyl radical scavenging effect as reported by Guo et al. [86].

In addition to Se, iron was also evidenced to correlate with the antioxidant actions of polysaccharides. Abu et al. [87] found that ascophyllan and fucoidan naturally contain certain amount of iron and other metal elements. After treatment with EDTA and subsequent dialysis, most of the metal elements except Mg were removed from these two polysaccharides, especially more than 90% of ferrous ions. EDTA treatment leads to a significant increase of ferrous chelating efficiency for both of the two polysaccharides with different extents and the effect may possibly depend on the inherently existent Fe levels in the polysaccharides.

### 3.5. Chemical Modification

Chemical modifications, such as sulfation, carboxymethylation, phosphorylation, benzoylation, acetylation, and NaIO_4_ oxidation, are evidenced to influence the antioxidant activity of polysaccharides to some extent. It is widely accepted that chemical modifications could enhance the antioxidant activity of polysaccharides, for example, sulfated polysaccharide from fresh persimmon (*Diospyros kaki* L.) fruit [88],* Tremella fuciformis* [89], acetylated, phosphorylated, and benzoylated levan-type exopolysaccharide from* Paenibacillus polymyxa *EJS-3 [90], phosphorylated polysaccharide from* Radix hedysari* [91], and acetylated and benzoylated derivatives from* Ulva pertusa* [92], which exhibited obviously stronger scavenging activity and/or reducing power than the unmodified polysaccharides. One mechanism is that the introduction of these substitution groups into polysaccharide molecules leads to weaker dissociation energy of hydrogen bond. Therefore, the hydrogen donating ability of polysaccharide derivatives was increased. Another mechanism is speculated to activate the abstraction of the anomeric carbon. On the other hand, the chemical modification is sometimes accompanied with a decrease of molecular weight, hence improving the antioxidant potentials of polysaccharides. Among the derivatives, the sulfate polysaccharide is commonly reported as a stronger antioxidant, which is partly due to its ordered, extended structure. The sulfated polysaccharide usually traps free radicals in an electrostatic manner since the sulfate groups usually generate a highly acidic environment and the sulfur substitution may also weaken hydrogen bond interactions between polysaccharides.

An algal sulfated polysaccharide, fucoidan, extracted from* Laminaria japonica* has been fully studied on various molecular modification derivatives. Six low molecular fucoidan derivatives (sulfated DFPS, acetylated ADF, benzoylated PHDF, phosphorylated PDF1 and PDF2, and aminated NDF) were all exhibiting potent antioxidant potentials. PHDF had the strongest radical scavenging abilities and DFPS had the highest reducing power [93]. Feng et al. [94] found unsulfated lentinan nearly detected antioxidant capacity, but when it was sulfated by either conventional heating or novel microwave radiation, the antioxidant effect was considerably enhanced, indicating the positive correlation between antioxidant effects and introduction of the sulfate group. Chen et al. [95] prepared four phosphorylated polysaccharides (POP1-p) from* Portulaca oleracea* L. and compared the antioxidant activity with the native POP1. They found that POP1-p had stronger scavenging effect on hydroxide radical, superoxide radical, and DPPH radical, as well as a higher ferrous ions chelating ability and reducing power.

Types of substitution groups and degrees of substitution (DS) appeared to have an effect on the physicochemical properties and conformation of native polysaccharides, such as molecular weight, polarity, solubility, and charge density. DS may also affect the activity through interruption of inter- and intramolecular hydrogen bonds. Chen et al. [96] found that not only did the total sugar content of acetylated and carboxymethylated derivatives decrease significantly, but also its molecular weight was reduced in contrast to native* G. atrum* polysaccharide. Liu et al. [97] proved that sulfation effectively improved the water solubility and bile acid-binding capacities of a water-insoluble polysaccharide from* G. lucidum* (GLP). Furthermore, ^13^C NMR results showed that C-2, C-4, and C-6 position might be partially substituted, and C-4 was the most reactive. It was probably due to its special structure features and the influence of steric hindrance.

A linear relationship between the degree of substitution and antioxidant potentials was not always observed, suggesting high DS was not necessary for antioxidant behavior. Xie et al. [98] revealed that antioxidant activity of sulfated CP with a highest DS of 0.55 was not as effective as derivatives with middle DS (0.42 and 0.06). However, the influence of DS was still disputable, as a high DS could enhance the antioxidant activity evidenced in many reports. Yan et al. [99] pointed out that the sulfation of exopolysaccharide, produced by* Cordyceps sinensis* fungus (Cs-HK1), occurred most frequently at hydroxyl groups of C-6 and caused a conformation change from random coils or aggregates to single helices in aqueous solution. The antioxidant activity of the sulfated derivatives for hydroxyl radical and ABTS radical scavenging effect was significantly enhanced with increasing DS and reducing molecular weight. Wang et al. [100] showed that C-6 substitution was predominately in phosphorylated derivatives of galactomannan (PGG) from guar gum according to ^13^C NMR analysis and PGG with high DS achieved a higher radical scavenging effect and stronger chelating ability than PGG with lower DS. Jung et al. found that DPPH radical scavenging ability of polysaccharide from* Pleurotus eryngii* was improved with increasing degree of sulfation [101]. This finding was also consistent with the report that high degree of sulfated substitution (0.90) was more effective than that of low DS (0.43) in scavenging DPPH [102]. Another study also revealed a positive relationship between the degrees of acetylated substitution and scavenging effects against DPPH and superoxide radical, as well as reducing power [103].

It is worth pointing out that the reducing power of polysaccharide was nearly lost after phosphorylated modification in some studies [104, 105]. This may be because the negative charged phosphorylate groups were effective at some specific sites of residues in certain polysaccharides, but they were weakened at others.

### 3.6. Structural Features of Polysaccharide

It is widely believed that the bioactivity of polysaccharides is affected by their structure characteristics, such as chemical composition, molecular mass, types of glycosidic linkage, and conformation. Differences in origin materials, extraction procedures, and even drying technologies that influence the physiochemical properties, structure, or conformation of polysaccharides will lead to differences in antioxidant activity, speculating their possible relationships [106–109]. Specifically, a correlation between molecular weight and radical scavenging activity was well documented [48, 57] and a similar observation in uronic acid content was reported as well in several reports [110, 111]. Additionally, it is suggested that the overall radical scavenging ability was associated with the number of hydroxyl or amino groups in polysaccharide molecules such as chitosan [112].

Molecular weight was one of the most important structural features of polysaccharide. A number of reports suggested that the antioxidant potency is mainly associated with molecular weight of polysaccharides. It was supposed that polysaccharides with low molecular weights would have more reductive hydroxyl group terminals (on per unit mass basis) to accept and eliminate the free radicals. Liu et al. [113] obtained two low molecular weight polysaccharides (GLP_L_1 and GLP_L_2) from* G. lucidum* and investigated the antioxidant activity of these two polysaccharides. Results showed that both GLP_L_1 and GLP_L_2 are effective radical scavenger and ferrous chelator. Xing et al. [114] reported that scavenging effect against superoxide radical of low molecular weight chitosan (9 kDa) was more potent than that of the high molecular one (760 kDa). The possible mechanism may be related to the structure characteristics of chitosan, which contained two hydrogen groups and one amino group in each monomer unit. High molecular weight chitosan has a more compact structure, resulting in stronger intramolecular hydrogen bond and thus making the hydrogen and amino groups restricted. By a stepwise fractionated precipitation with ethanol at a final concentration of 40%, 60%, and 80%, Zha et al. [115] obtained three polysaccharides from rice bran with a molecular weight ranging from 1.2 × 10^5^ to 6.3 × 10^6^ Da (PW1), 3.5 × 10^4^ to 7.4 × 10^4^ Da (PW2), and 5.3 × 10^3^ to 2.3 × 10^4^ Da (PW3), respectively. Results showed that PW3 exhibited the best potentials of reducing power, chelating metal ion, and scavenging abilities against DPPH and ABTS radical among three fractions, revealing that a relative low molecular weight fraction had high antioxidant abilities. Likewise, similar findings were also reported on other plant derived polysaccharides or extracellular polysaccharides [116–119].

Many techniques and methods (physically, chemically, or enzymatically) were used for degrading polysaccharides, resulting in a reduction of molecular weight, proved to influence the antioxidant activities as well. Feng et al. [120] revealed that a *γ*-ray treated chitosan had more pronounced antioxidant properties without changes in its backbone structure except for a decrease of molecular weight. Two well-known seaweeds polysaccharides, fucoidan and laminarin, were shown to increase the DPPH radical scavenging activity and reducing power after *γ*-ray irradiation, which result in the decrease of molecular weight and increase of carboxyl and carbonyl groups and double bonds [121].

Ultrasonic treatment, another method for degradation of polysaccharide, was also shown to enhance hydroxyl and superoxide anion radical scavenging capacity, chelating iron ion ability, and reducing power, possibly attributing to the decreased molecular weight and increased sulfate groups [122]. Zhang et al. [123] chemically degraded a polysaccharide from* Enteromorpha linza *using combination of ascorbic acid and H_2_O_2_ in order to obtain a lower molecular weight fraction, possessing higher hydroxyl scavenging effect and reducing power. Sun et al. [124] investigated the microwave-degraded polysaccharides from* Porphyridium cruentum* and found that high molecular weight polysaccharides had no obvious antioxidant activity, whereas the low molecular weight fragments showed strong scavenging effect on free radicals. And this microwave treatment did not apparently change the chemical components of the polysaccharide confirmed by physicochemical analysis.

However, inconsistent findings were also described. Cheng et al. [125] investigated the antioxidant potentials of polysaccharides from* Epimedium acuminatum*. Fractions with higher molecular weight displayed better antioxidant actions with regard to hydroxyl radical, H_2_O_2_-induced hemolysis inhibition, and lipid peroxidation. Additionally, Kardošová and Machová [126] revealed the effect of molecular weight was not significant based on similar antioxidant levels of polysaccharides and oligosaccharides.

Generally, the acidic polysaccharides, which contained a certain amount of uronic acid, were potent antioxidants [127, 128]. Therefore, uronic acid is considered to be another important indicator reflecting the antioxidant activity of the polysaccharides. It is supposed that the presence of electrophilic groups like keto or aldehyde in acidic polysaccharide facilitates the liberation of hydrogen from O-H bond. Evaluation of *β*-carotene linoleate emulsion ability, DPPH radical scavenging effect, and FRAP on glucuronic acid, galacturonic acid, and polygalacturonic acid was carried out in order to conclusively confirm the role of uronic acid in antioxidant potency [129]. Results showed that all the three kinds of uronic acid exhibited strong antioxidant effect in the order of polygalacturonic acid > glucuronic acid > galacturonic acid, indicating degree/nature of polymerization may impart the activity. But other compounds that also contained carboxylic group, such as formic, acetic, propionic, butyric, succinic, and citric acids, showed very low effect. The carbonyl group in the above acid was in an open chain, while in phenolic acid or uronic acid it was attached to a ring molecule. Li et al. [130] reported that two polysaccharide fractions (ZSP3c and ZSP4b) attained from* Zizyphus Jujuba *cv.* Jinsixiaozao* with the higher uronic acid content (25.5% and 29.0%, resp.) showed stronger free radical scavenging activities than ZSP1b containing no uronic acid. Different drying methods, such as hot air drying, vacuum drying, and freeze drying, will influence the antioxidant activity of polysaccharides. Among them, freeze drying was an appropriated and effective method to yield polysaccharides with higher free radical scavenging ability, reducing power and Fe^2+^-chelating ability, and the different contents of uronic acid might partly be involved in [131, 132]. Therefore, the antioxidant properties of polysaccharides might due to, in part, the presence of uronic acid.

Among various antioxidative molecules, sulfated polysaccharides effectively scavenge free radicals, bind metal ion catalysts to inhibit the continuous production of radicals, and protect against lipid peroxidation. Polysaccharide obtained from marine algae is a kind of native sulfated polysaccharide displaying considerable antioxidant activities. Yang et al. [133] compared the antioxidant activity of sulfated polysaccharide from* Corallina officinalis* and its desulfated derivatives. Results showed that the native sulfated polysaccharides possessed more excellent radical scavenging activity and reducing power than the desulfated fractions. The reduced antioxidant capacity after desulfated treatment was also evidenced on sulfated polysaccharides from* Undaria pinnatifida *[134]. Apart from molecular modification treatment, the native sulfated polysaccharide obtained from* Laminaria japonica* was demonstrated to be an effective antioxidant, partly related to the sulfate groups in the polysaccharide although other factors, that is, molar ratio of sulfate/(fucose or total sugar), molecular weight, could not be ignored as well [135]. Analysis of four sulfated polysaccharide fractions with different molecular weights prepared from* Ulva pertusa *Kjellm. demonstrated that lower molecular weight polysaccharide fractions, presented higher number of reducing and nonreducing ends, showed the stronger reducing power [136]. Pectic acids, known as polygalacturonic acids, showed extremely the highest reducing power among the tested polysaccharides, including chitosans and alginates with low and high molecular weights. Consequently, certain structural characteristics specific to the polysaccharides other than molecular weight might be responsible for the reducing power. Chitosan is a deacetylation product of chitin, which was naturally existent in the shells of crabs, shrimp, and krill. Both hydroxyl group and amino groups in chitosan backbone affect its antioxidant ability [137].

Except for the polysaccharide with anionic or cationic functional groups, such as chitosan, sulfated or phosphorylated glucans, most carbohydrates are not a class of potent antioxidant, significantly weaker than the synthetic antioxidants such as BHT, Trolox, and pyrrolidine dithiocarbamate. Chen et al. [138] have demonstrated that only polysaccharide in a polyelectrolyte form exhibited powerful antioxidant activities, that is, agar with sulfate group and chitosan with amino group, but not starch which only had hydroxyl group. Rao and Muralikrishna [129] revealed that neither glucose nor soluble starch, laminarin, showed any antioxidant activity according to the results of emulsion assay, even at very high concentration of 2 mg/mL. *β*-glucans from yeast cell walls appeared to exhibit a low antioxidant activity compared to other cell wall fractions, that is, protein [139, 140]. Another report [141] revealed that all yeast mannans (derived from* S. cerevisiae*,* C. dubliniensis*,* C. tropicalis*,* C. albicans* ser A, and* C. albicans *ser B) and commercial *β*-glucans (laminarin, lichenan, and curdlan) were weak DPPH and hydroxyl radical scavenger and poor Fe^2+^-chelator with the most effective one chelated only about 13.1% of Fe^2+^.

One proposed model for free radical scavenging effect was to subtract the anomeric hydrogen from carbohydrates by free radicals and combine it to form a neutral molecular [142], and then the generated alkoxyl radical promoted the intramolecular hydrogen abstraction reaction which triggers spirocyclization reaction to terminate the reaction of radical chain [143–145]. Further verification experiments to confirm the model, however, are not conducted.

On the other hand, the definite role of monosaccharide or glycosidic linkages in antioxidant activity of polysaccharide remained confused. Lo et al. [146] investigated the relationship between antioxidant properties of polysaccharides and monosaccharide or glycosyl linkages using four conventional antioxidant models (conjugated diene, reducing power, DPPH scavenging, and ferrous ions chelating) on multiple linear regression analysis (MLRA). Results revealed that compositions and ratios of monosaccharide as well as types of glycosyl linkages would be of concern in modulating the antioxidant properties. Specifically, rhamnose and mannose showed positive coefficients in all the four MLRA models. Meanwhile, glycosyl linkages, specifically arabinose 1→4 and mannose 1→2 of the side chain, were significantly related to the reducing power, whereas glucose 1→6 and arabinose 1→4 were closely in relation to DPPH radical scavenging effect. Tsiapali et al. [147] pointed out that a portion of antioxidant ability of the carbohydrate appeared to correlate with the monosaccharide composition rather than types of intrachain linkages, molecular weight, or degree of branching, since either dextrose or mannose showed weaker free radical scavenging ability than the polymer. It was interesting to find that the polymer had better radical scavenging effect than either of the monosaccharides, suggesting that the polymeric structure conferred additional activity of the carbohydrates. Meng et al. [148] adopted Pearson correlation analysis test and linear regression analysis to explore the relationship between the monosaccharide composition of polysaccharides and the antioxidant activity. Results showed that the antioxidant activity was significantly correlated with the content of mannose (*P* < 0.01) and glucose (*P* < 0.05), whereas galactose was not correlated (*P* > 0.05). Furthermore, both the contents of monosaccharide were observed to have high correlation coefficients concerning radical scavenging activity with mannose the positive (*r* = 0.942) and glucose the negative (*r* = −0.905).

## 4. Conclusion and Perspective

According to the extensive* in vitro *antioxidant studies, the polysaccharide is indeed an effective antioxidant. The underlying mechanism is, however, uncertain as the relationships between antioxidant activity and physicochemical properties or structural features are not comprehensively elucidated and confirmed. Besides, it is worth noting that conflicting results are observed in comparison with a number of literatures, since different sources, extraction methods, and even drying procedures can influence the evaluated polysaccharides. On the other hand, limited information is available on antioxidant activity of high-purity polysaccharides, and therefore other antioxidant substances, for instance, protein, peptide, and polyphenol, that may always be retained in polysaccharides in a form of either conjugation or mixture should be taken into account. Overall, the antioxidant potentials of polysaccharides are not determined by a single factor but a combination of several related factors.

In the future, more studies should be concentrated on the exact mechanism of* in vitro* antioxidant activities of polysaccharides themselves and design some experiments if pure carbohydrates possess* in vitro* antioxidative capacity. Based on the various antioxidant mechanisms, different antioxidative evaluations, such as DPPH radical scavenging, hydroxyl radical scavenging, ABTS radical scavenging, reducing power, and chelating ability, should be adopted to assess the respective influences of carbohydrates, especially their structural features. With regard to the intensive researches on natural antioxidants, the properties of polysaccharides from natural products useful for the antioxidant activity and healthy benefits should be clearly elucidated, and more specific research, therefore, on this topic is imperative.
